# Carcinogenic effects of ptaquiloside in bracken fern and related compounds

**DOI:** 10.1054/bjoc.2000.1368

**Published:** 2000-09-04

**Authors:** D M Potter, M S Baird

**Affiliations:** Menai Organics Ltd, MENTEC, Deiniol Road, Bangor, Gwynedd LL57 2UP; Department of Chemistry, University of Wales, Bangor, Gwynedd, LL57 2UW, UK

**Keywords:** carcinogenic, ptaquiloside, bracken, ferns, illudins

## Abstract

Consumption of the bracken fern *Pteridium aquilinum* by cattle has been shown to induce bladder and intestinal carcinomas in cattle and to cause a number of diseases in other farm animals. An unstable glucoside named ptaquiloside, containing a reactive cyclopropane ring, has been isolated from the fern and its potent carcinogenicity proven. Nineteen of 31 ferns tested by chemotaxonomic methods in Japan have been found to contain potentially carcinogenic ptaquilosides as have *Cheilanthes sieberi* and *Pteridium esculentum*. Hydrolysis of ptaquilosides leads to pterosins; under milder conditions a dienone which is believed to be the primary carcinogen is obtained. Hypacrone, a sesquiterpine containing a reactive cyclopropane ring, has been isolated from *Hypolepis punctata* and its structure proved by synthesis. Illudins, structurally similar to ptaquiloside, have been isolated from the basidiomycete *Omphalotus illudens*. These give anti-tumour activity and similar reactivity with nucleophiles to ptaquiloside. Compound CC-1065, a highly toxic antibiotic also containing a cyclopropane ring, has been isolated from *Streptomyces zelensis*. The mechanism of its reactivity with DNA has been compared to that of ptaquiloside and the small structural differences between carcinogenic and anti-tumour activity discussed. Both CC-1065 and adozelesin, a synthetic analogue with anti-tumour activity, have been shown to alkylate the N-3 atom of adenine in a certain sequence of DNA. The reactivity of cysteine with ptaquilosides and illudins is discussed, as is the role of cysteine alkylating agents in apoptosis. © 2000 Cancer Research Campaign


*British Journal of Cancer* (2000) **83**(7), 914–920
© 2000 Cancer Research Campaign
doi: 10.1054/ bjoc.2000.1368, available online at http://www.idealibrary.com on IDEAL®

### Carcinogenic effects of ptaquiloside in bracken fern and related compounds

**DM Potter[1] and MS Baird[2]**

Menai Organics Ltd, MENTEC, Deiniol Road, Bangor, Gwynedd, LL57 2UP; [2]Department of Chemistry, University of Wales, Bangor, Gwynedd, LL57 2UW, UK

**Summary** Consumption of the bracken fern *Pteridium aquilinum* by cattle has been shown to induce bladder and intestinal carcinomas in cattle and to cause a number of diseases in other farm animals. An unstable glucoside named ptaquiloside, containing a reactive cyclopropane ring, has been isolated from the fern and its potent carcinogenicity proven. Nineteen of 31 ferns tested by chemotaxonomic methods in Japan have been found to contain potentially carcinogenic ptaquilosides as have *Cheilanthes sieberi* and *Pteridium esculentum*. Hydrolysis of ptaquilosides leads to pterosins; under milder conditions a dienone which is believed to be the primary carcinogen is obtained. Hypacrone, a sesquiterpine containing a reactive cyclopropane ring, has been isolated from *Hypolepis punctata* and its structure proved by synthesis. Illudins, structurally similar to ptaquiloside, have been isolated from the basidiomycete *Omphalotus illudens*. These give anti-tumour activity and similar reactivity with nucleophiles to ptaquiloside. Compound CC-1065, a highly toxic antibiotic also containing a cyclopropane ring, has been isolated from *Streptomyces zelensis*. The mechanism of its reactivity with DNA has been compared to that of ptaquiloside and the small structural differences between carcinogenic and anti-tumour activity discussed. Both CC-1065 and adozelesin, a synthetic analogue with anti-tumour activity, have been shown to alkylate the N-3 atom of adenine in a certain sequence of DNA. The reactivity of cysteine with ptaquilosides and illudins is discussed, as is the role of cysteine alkylating agents in apoptosis. © 2000 Cancer Research Campaign

**Keywords**: carcinogenic, ptaquiloside, bracken, ferns, illudins

The bracken fern *Pteridium aquilinum* is among the most common plants (Page, 1976; Taylor, 1990). Ingestion by cattle of either the whole plant or extracts leads to a syndrome in which there is pannyeloid bone-marrow damage, pyrexia and often gut-lining damage and ulceration (Evans et al, 1958). Also typical are widespread petechelial haemorrhages (Evans et al, 1954). The presence of a carcinogen in fresh bracken ferns was first recognized over 30 years ago by Evans and Mason (1965) in trials on mice and later confirmed by other workers (Pamacku and Price, 1969; Hirono et al, 1970). Consumption of bracken fern by cattle has been shown to induce bladder and intestinal carcinomas (Fenwick, 1965; Smith, 1990) and to cause a number of diseases in other farm animals (Evans, 1979; 1986). The presence of haematuria in cattle fed bracken over long periods has been reported, together with changes of a polypous-tumerous nature in the bladder mucosa (Rosenberger, 1960). When introduced into the urinary bladders of dogs, an extract of urine from cattle fed hay in haematuria districts produced changes similar to haemangionoma. Application of the same extracts to the skin of white mice led to papilloma-type excrescences (Georgiev et al, 1963). Bracken has been identified as a farm hazard of considerable economic importance (Alonso-Amelot et al, 1995). Chemical studies of over thirty species of Pteridaceae have been carried out (Murakami and Tanaka, 1988); an unstable glucoside named ptaquiloside (Figure 1A) was isolated (Niwa et al, 1983; Van der Hoeven et al, 1983) and its potent carcinogenicity proven (Hirono et al, 1984). Ptaquiloside was shown to break down in aqueous solution in the presence of acid, base or heat to give pterosin B (Figure 2A, $R_1 = CH_2OH$, $R_3 = H$). As well as the above carcinogenic effects, other compounds present in bracken fern cause a variety of effects such as cyanogenesis and thiamine deficiency (Smith, 1997).

Three main routes may lead to human exposure to the toxic effects of bracken fern – eating the plant, physical contact with it or more particularly the spores, and ingestion of milk from affected animals. The consumption of the plant is common in Japan and, although pre-treatment with boiling water or soda ash reduces the carcinogenicity, the risk of oesophageal cancer is increased approximately by 2.1 in men and 3.7 in women (Kamon and Hirayama, 1975). Ptaquiloside has been identified in the milk produced by bracken-fed cows (Alonso-Amelot et al, 1993; 1996); the concentration in milk has been found to be about 8.5% of the amount ingested by the cow and is linearly dependent on the dose. The authors indicate that, in their view, it is ‘certainly likely’ that ptaquiloside in milk is responsible for the connection between bracken infestation and the incidence of gastric cancer in populations of farmers inhabiting cattle-range areas in Costa Rica and other countries where bracken growth is dense (Villalobos-Salazar, 1985), strongly suggesting the need to avoid the distribution of this into the food chain. Indeed neoplasia has been caused by feeding rats with the milk of cows or rats fed on a bracken fern diet (Evans et al, 1972; Villalobos-Salazar et al, 1990). A possible spatial association between bracken and cancer in England and Wales has been examined (Wells and McNally, 1989), while aerial exposure to spores may be a problem given the development of neoplasia in mice treated with spores (Evans et al, 1986; Villalobos-Salazar et al, 1995) and the observation that this treatment leads to DNA adducts in upper gastrointestinal tissues (Povey et al, 1996).

*Received 4 January 2000*
*Revised 18 May 2000*
*Accepted 22 May 2000*

*Correspondence to:* DM Potter

This paper originated from the IVth International Bracken Fern Conference (20–23 July 1999)

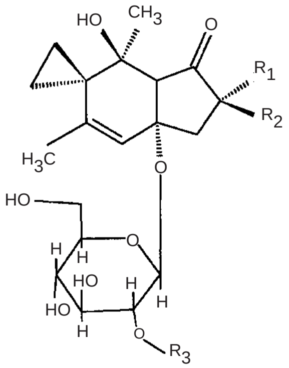

| | $R_1$ | $R_2$ | $R_3$ |
|---|---|---|---|
| **A** | $CH_3$ | H | H |
| **B** | $CH_2OH$ | $CH_3$ | H |
| **C** | $CH_3$ | $CH_3$ | H |
| **D** | $CH_3$ | $CH_3$ | acetyl |
| **E** | $CH_2OH$ | $CH_3$ | acetyl |
| **F** | H | $CH_3$ | H |

**Figure 1** Structure of (**A**) ptaquiloside, (**B**) caudatoside, (**C**) methylptaquiloside, (**D**) hypoloside A, (**E**) dentoside A and (**F**) isoptaquiloside

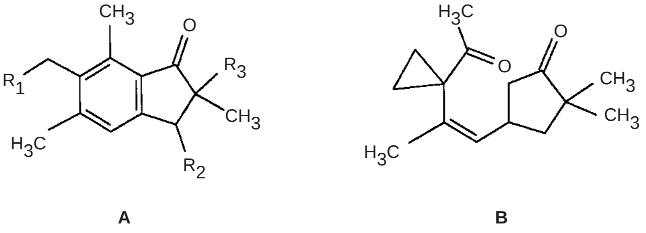

**Figure 2** (**A**) Pterosin B and (**B**) hypacrone

**Table 1** Distribution of the ptaquilosides in ferns (Saito et al, 1989; Niwa et al, 1983)

| Species | Ptaquiloside or ptaquiloside analogue |
|---|---|
| Cheilanthes myriophylla | + |
| Cibotium harometz | + |
| Coniogramme gracilis | – |
| C. intermedia | – |
| C. japonica | – |
| Dennstaedtia distenta | – |
| D. hirsta | + |
| D. scabra | + |
| Histiopteris incisa | + |
| Hyplepis bamleriana | + |
| H. tenuifolia | + |
| H. punctata | + |
| Microlepia marginata var. hipinnata | – |
| M. strigosa | + |
| Monachosorum arakii | – |
| Monachosorum flagellare | – |
| Onychium japonicum | + |
| Pityrogramma calomelanos | + |
| P. sulphurea | + |
| Pteridium aquilinum | + |
| Pteris cretica | + |
| P. dispar | + |
| P. excelsa | + |
| P. fauriei | – |
| P. nipponica | + |
| P. oshimensis | + |
| P. purpureorachis | – |
| P. ryukyuensis | – |
| P. semipinnata | – |
| P. tremula | + |
| P. wallichiana | + |
| Sphenomeris chusana | – |

(+ corresponds to present, – to not detected)

### The distribution and structure of ptaquilosides and pterosins

The distribution of ptaquiloside in a variety of ferns has been examined using chromatography and a modified Ames test (Saito et al, 1989). Nineteen of the 31 ferns tested were found to contain the potentially carcinogenic ptaquilosides. They are listed in Table 1. Variable concentrations are present, but these can be as high as 1.3% of the dry weight (Smith et al, 1992; 1994).

Many species of fern have been found to contain derivatives of pterosin B or related pterosins (Figure 2A). The structure of some of these are shown in Table 2 (Fukuoka et al, 1978; Ng and McMorris, 1984). *Pteridium aquilinum* has been found to contain 20 kinds of pterosins and pterosides but until recently only ptaquiloside yielding pterosin B on hydrolysis has been characterized (Saito et al, 1989). It was expected that ptaquiloside analogues would be present in *Pteridium aquilinum*, which would on hydrolysis yield pterosin Z and pterosin A. The ptaquiloside (Figure 1A), precursor to pterosin A, has recently been isolated from *Pteridium aquilinum* var. *caudatum* (Castillo et al, 1997), as has an epimer of ptaquiloside, isoptaquiloside (Figure 1F) while the precursor (Figure 1C) of pterosin Z has been isolated from *Pteridium aquilinum* subsp. *aquilinum* (Potter and Pitman, 1994). This precursor of pterosin Z has also now been isolated from *Pteridium aquilinum* var. *caudatum* (Castillo et al, 1998).

*Cheilanthes sieberi*, growing in both Australia and New Zealand, had been found to cause a syndrome indistinguishable from acute bracken poisoning; *Cheilanthes sieberi* fronds and croziers collected from both Australia and New Zealand were found to contain ptaquiloside (Smith et al, 1990; Agnew and Lauren, 1991). Analysis of the extracts for pterosin B following base and acid treatment gave concentrations twice as high as expected for the ptaquiloside alone, possibly suggesting pterosin B precursors other than ptaquiloside in *Cheilanthes sieberi* (Smith et al, 1990).

Further analogues of ptaquiloside have been isolated from other ferns including hypolosides A, B and C from *Hypolepis punctata* and B and C from *Dennstaedtia hirsta* (Saito et al, 1990), from which dennstoside A (Figure 1E) was also isolated (Koyama et al, 1991). Hypolosides B and C have the same structure as hypoloside A (Figure 1D), but with an additional p-coumaroyl group on the 4-position of the glucose. The only difference between hypolosides B and C is that they are geometrical isomers with the p-coumaroyl group existing in the *cis* and *trans* isomers respectively. Hypoloside A, B and C also yield pterosin Z on hydrolysis and dennstoside A yields pterosin A. Extraction of fresh shoots of *Hypolepis punctata* yielded hypacrone (Figure 2B) (Hayashi, 1977*a*; 1977*b*), also possessing a reactive cyclopropane ring and giving pterosin Z on hydrolysis.

*© 2000 Cancer Research Campaign*

**Table 2** Structures of some of the fern pterosins (Figure 2A)

| Compound | Pterosin | R[1] | R[2] | R[3] |
|---|---|---|---|---|
| a | B | $CH_2OH$ | H | H |
| b | O | $CH_2OCH_3$ | H | H |
| c | F | $CH_2Cl$ | H | H |
| d | E | $CO_2H$ | H | H |
| e | Palmityl | | | |
| | Pterosin B | $CH_2O$ palmityl | H | H |
| f | Pterosin B | $CH_2O$ glucose | H | H |
| g | Pterosin C | $CH_2OH$ | OH | H |
| h | J | $CH_2Cl$ | OH | H |
| i | Acetylpterosin | | | |
| | C | $CH_2OAc$ | OH | H |
| j | Pteroside C | $CH_2OGlu$ | OH | H |
| k | I | $CH_2OCH_3$ | H | $CH_3$ |
| l | Z | $CH_2OH$ | H | $CH_3$ |
| m | H | $CH_2Cl$ | H | $CH_3$ |
| n | D | $CH_2OGlu$ | OH | $CH_3$ |
| o | Pteroside D | $CH_2OGlu$ | OH | $CH_3$ |
| p | A | $CH_2OH$ | H | $CH_2OH$ |
| q | K | $CH_2Cl$ | H | $CH_2OH$ |
| r | L | $CH_2OH$ | H | $CH_2OH$ |
| s | V | $CH_2OCH_3$ | H | $CH_2OH$ |

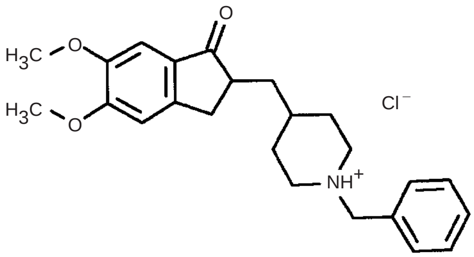

**Figure 3** Donepezil (Gopinath 1992)

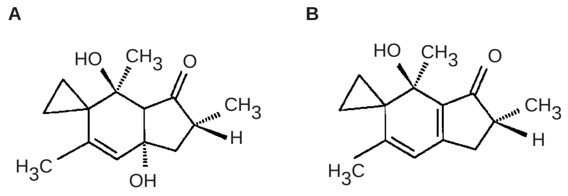

**Figure 4** (**A**) Ptaquilosin, (**B**) Unstable dienone

### Biological activity of pterosins and related indanone structures

Pterosins, which not only are widely occurring in ferns (Hayashi et al, 1972; Fukuoka et al, 1978; Ng and McMorris, 1984), but also in certain fungi of the class Basidiomyces including *Fomes annosus* and *Cyathus bulleri*, have been found not to possess carcinogenic activity, although some show cytotoxicity to HeLa cells (Yoshihira et al, 1978). Several pterosins produced cytotoxic effects on the ciliate, *Paramecium caudatum* and also induced abnormal development of urchin embryos (Kobayashi and Koshimizu, 1980). Pterosin B and O have been isolated from *Pteris inequalis* and shown to be active against *Bacillus subtilis* (Kobayashi et al, 1975). Recently, a substituted indanone, structurally similar to pterosins, has been approved for the treatment of Alzheimer's disease in the USA and UK (Gopinath, 1998). This compound, developed by Eisai and co-marketed by Pfizer has the structure shown in Figure 3.

The binding-site of this compound in acetylcholinesterase (ACE) has been investigated and possible structural modifications for identifying improved ACE inhibitors for the treatment of Alzheimer's disease discussed (Pang and Kozikowski, 1994).

### Biological activity of ptaquilosides and related structures

Under weakly alkaline conditions both ptaquiloside and its aglycone ptaquilosin (Figure 4A) are converted, with D-(+) glucose liberation in the former case, into the unstable dienone (Figure 4B), which is the activated form regarded as the ultimate carcinogen (Kigoshi et al, 1993). Both the ileum and the urine of herbivores are known to be alkaline. In the field, cattle and sometimes sheep grazing on bracken fern develop tumours, most frequently in the urinary bladder. The $^{32}$P-postlabelling assay has provided evidence for ptaquilosin-DNA adducts in the ileum of bracken fern-fed calves. Alkylation occurred primarily at the

*© 2000 Cancer Research Campaign*

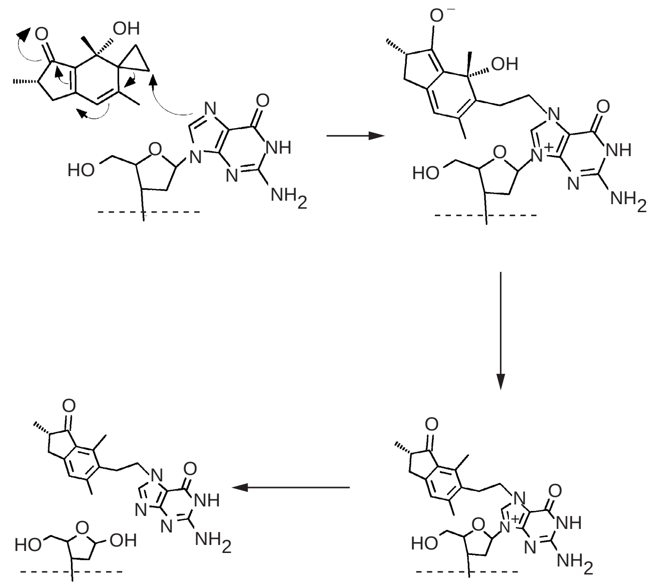

**Figure 5** Part of the reaction of ptaquilosin and deoxytelranucleotide

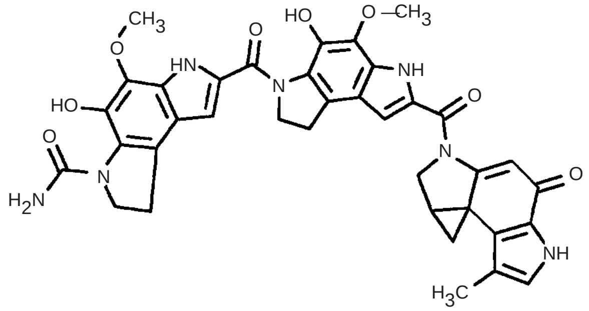

**Figure 6** Compound CC-1065

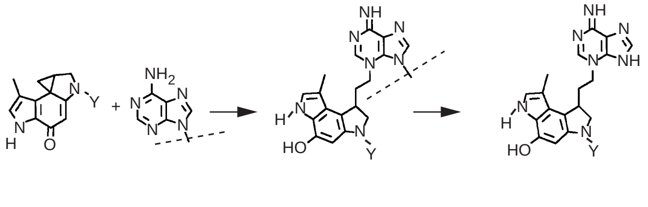

**Figure 7** Reaction of CC-1065 with DNA

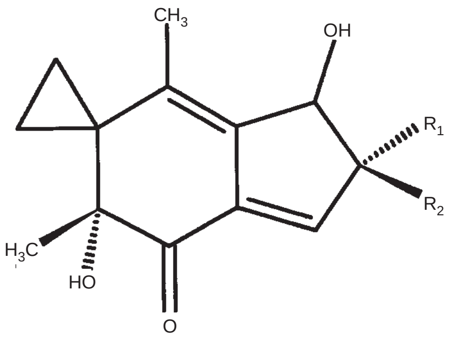

**Figure 8** Illudin S ($R_1$ = $CH_2OH$, $R_2$ = $CH_3$) and illudin M ($R_1$ = $R_2$ = $CH_3$)

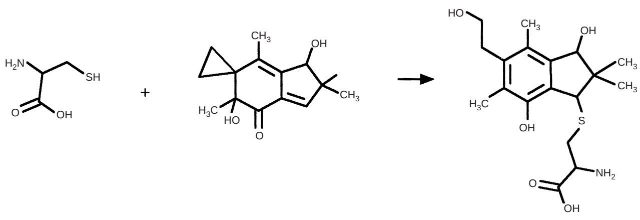

**Figure 9** The reaction of cysteine with illudin M

adenine groups; the results suggest that initial alkylation of adenine by ptaquilosin (ptaquiloside) in codon 61 followed by depurination and error in DNA synthesis leads to activation of H-*ras* proto-oncogene (Prakash et al, 1996).

Ptaquilosin is strongly electrophilic and reacts readily with amino acids, nucleosides and nucleotides (Ojika et al, 1987) under mild conditions, forming covalent adducts with DNA and causing DNA strands to break (Ojika et al, 1989). In a model reaction with a deoxytetranucleotide, part of which is shown in Figure 5, the covalent adduct was found at a guanine residue and a corresponding adduct was found at an adenine residue.

Compound CC-1065, a highly toxic antibiotic containing a reactive cyclopropane ring, the structure of which is shown in Figure 6, has been isolated from *Streptomyces zelensis* (Hanka et al, 1978; Kushida et al, 1994). This compound shows significant cytotoxicity in vitro and anti-tumour activity in vivo (Kushida et al, 1994) and is significantly more toxic than actinomycin, vinblastine or maytanosine (Martin et al, 1978).

CC-1065 is believed to bind to DNA without intercalation (Swenson et al, 1981). DNA cleavage occurred through a similar mechanism to that for ptaquiloside, namely by formation of an

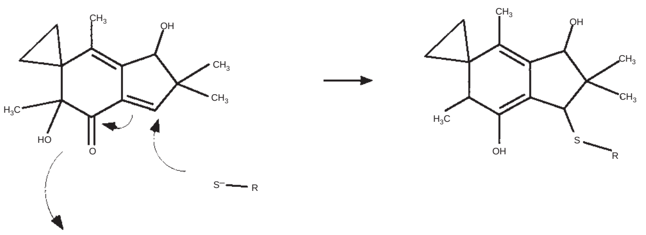

Figure 10 Reaction of illudin M with thiols

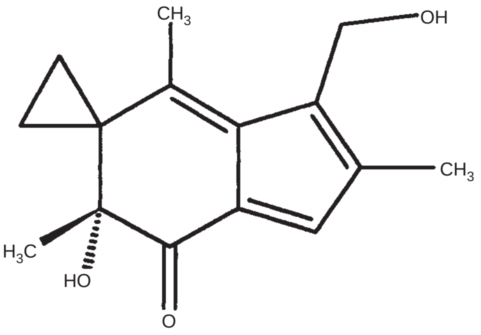

Figure 11 Hydroxymethylacylfulvene alkyladenine adduct by attack of the base on the cyclopropane in a homo-Michael addition, activated by the adjacent enone system and leading to the formation of an aromatic adduct, and then to depurination and strand breakage on heating (Figure 7) (Kushida et al, 1994). However, two significant differences have been observed. Ptaquiloside forms adducts at both guanine and adenine residues and induces the spontaneous cleavage at adenine base sites under physiological conditions, whereas CC-1065 only causes cleavage at higher temperature (70°C). At lower temperature (37°C) the adenine adduct of CC-1065 undergoes a retro-homo-Michael reaction to regenerate the initial CC-1065 structure and, it is presumed, intact DNA; the reverse reaction may require the double helical structure in DNA and be inhibited in melts (Warpehoski et al, 1992). At the same lower temperature ptaquiloside depurinates spontaneously. Adozelesin, a synthetic analogue of CC-1065 was developed with potent anti-tumour activity; both this compound and CC-1065 were found to alkylate the N-3 atom of adenine only in a certain sequence of DNA.

Illudins (Figure 8) structurally similar to ptaquiloside have been isolated from *Omphalotus illudens* and related fungi (Anchel et al, 1950; McMorris and Anchel, 1963; 1965) and have been found to be rapidly cytotoxic to haematopoietic tumours in vitro at extremely low concentrations (Kelner et al, 1987).

The illudins have been found to be quite reactive at low pH and to behave as bifunctional alkylating agents (McMorris et al, 1989). Unlike ptaquiloside, they have been found to react only with thiols including cysteine (Figure 9) at physiological pH (McMorris et al, 1990).

This reaction is thought to occur through initial attack of the thiolate at one of the enone systems in illudin to give an intermediate cyclopropane (Figure 10) which is then ring-opened by attack by solvent leading to a phenolic system (McMorris et al, 1990).

Illudin M and S failed to react with adenosine and other nitrogen nucleophiles and do not react directly with DNA. Many of these compounds are too toxic to find application, however hydroxymethylacylfulvene (McMorris, 1996) (HMAF) (Figure 11) causes complete tumour regression in all animals at the maximum tolerated dose, proving effective against breast, lung and colon cancers in animal models and in human cell cancer clones (McDonald, 1997). This compound is now available from a chemical synthesis (McMorris, 1997).

$^{32}$P-postlabelling analysis of DNA adducts formed in the upper gastrointestinal tissue of mice fed bracken extract or spores has shown that similar adducts are formed when two cyclopropane rings containing compounds 1-(4-chlorophenylsulphonyl)-1-cyclopropane and 3-cyclopropylindeno [1,2-c]pyrazol-4-(O-methyl)oxime are used instead (Povey et al, 1996). The cyclopropane rings of these compounds are also much more stable to reaction with nitrogen nucleophiles than ptaquiloside. Also it has been found that spirocyclopropane-4-piperidinones show moderate DNA cleaving activity, although again the cyclopropane rings are less strained and more stable than in ptaquilosides (Anichini et al, 1997). It is possible that these more stable cyclopropanes do not react directly with DNA. For example, the anti-cancer activity of Taxol, a compound extracted from yew trees, is mediated by tubulin polymerization and microtubule formation, thereby blocking the cell cycle in mitosis and inducing programmed cell death (apoptosis) (Wahl et al, 1996; Kinloch et al, 1999). Recently, apoptosis has become an area of intense scientific interest. This is the study of the triggers and mechanisms involved in cell death (Kinloch et al, 1999). A number of low-molecular-weight compounds have been shown to enhance or inhibit this fundamental cellular process. Decrease in the rate of apoptosis may facilitate the growth of tumours, while blocking a protein's anti-apoptotic function is of interest as potential drug targets for cancer treatment (Kinloch et al, 1999). Molecules containing a cyclopropane ring, such as ptaquilosides or the illudins, which react with thiol nucleophiles such as cysteine may act as cysteine–protease inhibitors, affecting the complex process of apoptosis giving either carcinogenic or anti-tumour activity without direct reaction with DNA. This warrants further investigation. Cysteine alkylating reagents such as N-ethylmaleimide and peptide aldehydes have been shown to be potent inhibitors of cysteine proteases inhibiting apoptosis (Nicholson et al, 1995). The control of apoptosis may lead to treatments for degenerative diseases such as Alzheimer's, Parkinson's and Huntington's disease, immune deficiency, leukaemias and other cancers.

### ACKNOWLEDGEMENTS

This paper originated from the IVth International Bracken Conference (20–23 July 1999).

© 2000 Cancer Research Campaign

### REFERENCES

Agnew PM and Lauren DR (1991) Determination of ptaquiloside in bracken fern (*Pteridium esculentum*). *J Chromatography* **538**: 462–468

Alonso-Amelot ME, Castillo U and De Jongh F (1993) On the passage of the bracken fern carcinogen, Ptaquiloside into bovine milk. *Le Lait* **73**: 323–332

Alonso-Amelot ME, Rodulfo-Baechler S and James-Espinosa R (1995) Comparative dynamics of Ptaquiloside and Pterosin B in the two varieties (*Caudatum* and *Arachnoideum*) of neotropical bracken fern (*Pteridium aquilinum* L. Kuhn). *Biochem Sysy Ecol* **23**: 709–716

Alonso-Amelot ME, Castillo U, Smith BL and Lauren DR (1996) Bracken ptaquiloside in milk. *Nature* **382**: 587

Anchel M, Hervey A and Robbins WJ (1950) Antibiotic substances for basidiomycetes VII *Clitocybe illudens*. *Proc Natl Acad Sci USA* **6**: 300–305

Anichini B, Goti A, Brandi A, Kuzhushkov SI and de Meijere A (1997) Versatile synthetic approaches towards aza-analogues of Illudin and Ptaquilosin sesquiterpenes. *Synlett* 25–26

Castillo UF, Wilkins AL, Lauren DR, Smith BL, Towers NR, Alonso-Amelot ME and Jaimes-Espinoza R (1997) Isoptaquiloside and caudatoside, illudane-type sesquiterpene glucosides from *Pteridium aquilinum* var. *caudatum*. *Phytochemistry* **44**: 901–906

Castillo UF, Ojika M, Alonso-Amelot M and Sakagami Y (1998) Ptaquiloside Z, a new toxic unstable sesquiterpene glucoside from the neotropical bracken fern *Pteridium aquilinum* var. *caudatum. Bioorganic and Medicinal Chemistry* **6**: 2229–2233

Evans IA (1979) Bracken carcinogenicity. *Res Vet Sci* **26**: 339–348

Evans WC (1986) In *Bracken Ecology, Land Use and Control Technology*, Smith RT and Taylor JA (eds), pp 121–132. Parthenon Publications: Carnforth, UK

Evans IA and Mason J (1965) Carcinogenic activity of bracken. *Nature* **208**: 913–914

Evans WC, Evans ETR and Hughes LE (1954) Studies on bracken poisoning in cattle. *Brit Vet J* **110**: 295, 365, 426

Evans WC, Evans IA, Thomas AJ, Watkins JE and Chamberlain AG (1958) Studies on bracken poisoning in cattle. *Brit Vet J* **114**: 180

Evans WC, Smith RT and Taylor JA (1986) The acute diseases caused by bracken in animals. In *Bracken Ecology, Land Use and Control Technology*, Smith RT and Taylor JA (eds), pp 139–146: Parthenon Publications: Carnforth, UK

Evans IA, Jones RS and Mainwaring-Burton R (1972) Passage of bracken fern toxicity into milk. *Nature* **237**: 107

Fenwick GR (1965) Bracken (*Pteridium aquilinum*) – toxic effects and toxic constituents. *J Sci Food Agric* **46**: 147–173

Fukuoka M, Kuroyanagi M, Yoshihira K and Natori S (1978) Chemical and toxicological studies on bracken fern, *Pteridium aquilinum* var. *latiusculum II*. Structures of pterosins, sesquiterpenes having 1-indanone skeleton. *Chem Pharm Bull* **26**: 2365–2385

Georgiev R, Vrigasov A, Antonov A and Dimitrov A (1963) Versuche zur Feststellung der Anwesenheit kanzerogener Stoffe im Harn der mit Heu aus Hamaturiegebieten gefutterten Kuhe. *Wiener Tierarztliche Monantsschrift* **196**: **50**(4): 589–595

Gopinath L (1998) Outsmarting Alzheimer's disease. *Chemistry in Britain* **34**: 38–40

Hanka LJ, Dietz A, Gerpheide SA, Kuentzel SL and Martin DG (1978) CC-1065 (NSC-298223), a new antitumour antibiotic. Production, *in vitro* biological activity, microbiological assays and taxonomy of the producing microorganism. *J Antibiotics* 1211

Hayashi Y, Nishizawa M, Harita S and Sakan T (1972) Structures and syntheses of Hypolepin A, B and C, sesquiterpenes from *Hypolepis punctata* METT. *Chem Lett* 375–378

Hayashi Y, Nishizawa M and Sakan T (1977*a*) Studies on the sesquiterpenoids of *Hypolepis punctata* METT.-I. *Tetrahedron* **33**: 2509–2511

Hayashi Y, Nishizawa M and Sakan T (1977*b*) Studies on the sesquiterpenoids of *Hypolepis punctata* METT.-II. *Tetrahedron* **33**: 2513–2519

Hirono I, Shibuya C, Fushimi K and Haga M (1970) Studies on carcinogenic properties of bracken (*Pteridium aquilinum*). *J Natl Cancer Inst* **45**: 179–188

Hirono I, Yamada K, Niwa N, Shizuri M, Ojika M, Hasaka S, Yamaji T, Wakamatsu K, Kigashi H, Nuyama K and Uosaki Y (1984) Separation of carcinogenic fraction of bracken fern. *Cancer Lett* **21**: 239–246

Kamon S and Hirayama T (1975) Epidemiology of cancer of the oesophagus in Mye, Nara and Wakayama prefecture with special reference to the role of bracken fern. *Proc Jpn Cancer Assoc* **34**

Kelner MJ, McMorris TC, Beck WT, Zamora JM and Taetle R (1987) Preclinical evaluation of illudins as anticancer agents. *Cancer Res* **47**: 3186–3189

Kigoshi H, Imamura Y, Mizuta K, Niwa H and Yamada K (1993) Total synthesis of natural (–)-ptaquilosin, the aglycone of a potent bracken carcinogen ptaquiloside, and the (+)-enantiomer and their DNA cleaving activities. *J Am Chem Soc* **115**: 3056–3065

Kinloch RA, Treherne JM, Furness LM and Hajimohamadreza I (1999) The pharmacology of apoptosis. *Trends Pharmacol Sci* **20**: 35–42

Kobayashi A, Egawa H, Koshimizu K and Mitsui T (1975) Antimicrobial constituents in *Pteris inequalis*. *Agric Biol Chem* **39**: 1851–1856

Kobayashi A and Koshimizu K (1980) Cytotoxic effects of bracken fern (*Pteridium aquilinum*) constituents, pterosins, on sea urchin (*Anthocidaris crassispina*) embryos and a ciliate (*Paramecium caudatum*). *Agric Biol Chem* **44**: 393–398

Koyama K, Takatsuki S and Natori S (1991) Dennstoside A, an analogue of Ptaquiloside, from *Dennstaedtia scabra*. *Phytochemistry* **30**: 2080–2082

Kushida T, Uesugi M, Sugiura Y, Kigoshi H, Tanaka H, Hirokawa J, Ojika M and Yamada K (1994) DNA damage by ptaquiloside, a potent bracken carcinogen: Detection of selective strand breaks and identification of DNA cleavage products. *J Am Chem Soc* **116**: 479–486

Martin DG, Hanka LJ and Neil GL (1978) Isolation, characterization and preliminary antitumour evaluation of CC-1065, a potent new agent from fermentation. *Proc Am Ass Cancer Res* **19**: 99

McDonald JR, Muscoplat CC, Dexter DL, Mangold GL, Chen S-F, Kelnar MJ, McMorris TC and von Hoff DD (1997) Preclinical antitumour activity of 6-hydroxymethylacylfulvene, a semisynthetic derivative of the mushroom toxin illudin S. *Cancer Res* **57**: 279–283

McMorris TC (1996) (Hydroxymethyl)acylfulvene, an illudin derivative with superior antitumour properties. *J Nat Prod* **59**: 896–899

McMorris TC (1997) Total synthesis of hydroxymethylacylfulvene, an antitumour derivative of illudin S. *J Chem Soc* (Chem. Comm.) **315**

McMorris TC and Anchel M (1963) The structures of the basidiomycete metabolites illudin S and illudin M. *J Am Chem Soc* **85**: 831

McMorris TC and Anchel M (1965) Fungal metabolites. The structures of the novel sesquiterpenoids illudin S and illudin M. *J Am Chem Soc* **87**: 1594–1600

McMorris TC, Kelner MJ, Chadha RK, Siegel JS, Moon S and Moya MM (1989) Structure and reactivity of illudins. *Tetrahedron* **45**: 5433

McMorris TC, Kelner MJ, Wang W, Moon S and Taetle R (1990) On the mechanism of toxicity of illudins: the role of glutathione. *Chem Res Toxicol* **3**: 574–579

Murakami T and Tanaka N (1988) Occurrence, structure and taxonomic implications of fern constituents. *Prog Chem Org Nat Prod* **54**: 1–353

Ng KE and McMorris T (1984) An efficient synthesis of pterosin C and other pterosins. *Can J Chem* **62**: 1945–1953

Nicholson DW, Ali A, Thornberry NA, Vaillancourt JP, Ding CK, Gallant M, Gareau Y, Griffin PR, Labelle M, Lazebnik YA, Munday NA, Raju SM, Smulson ME, Yamin T-T, Yu VL and Miller DK (1995) Identification and inhibition of the ICE/CED-3 protease necessary for mammalian apoptosis. *Nature* **376**: 37–43

Niwa H, Ojika M, Wakamatsu K, Yamada K, Hirono I and Matsushita K (1983) Ptaquiloside, a novel norsesquiterpene glucoside from bracken (*Pteridium aquilinum* var. *latiusculum*). *Tetrahedron Lett* **24**: 4117–4120

Ojika M, Wakamatsu K, Niwa H and Yamada K (1987) Ptaquiloside, a potent carcinogen isolated from bracken fern *Pteridium aquilinum* var. *latiusculum*: structure, elucidation based on chemical and spectral evidence, and reactions with amino acids, nucleosides and nucleotides. *Tetrahedron* **43**: 5261–5274

Ojika M, Sugimoto K, Okazaki T and Yamada K (1989) Modification and cleavage of DNA by Ptaquiloside. A new potent carcinogen isolated from bracken fern. *J Chem Soc* (Chem. Commun) 1775–1777

Page CM (1976) The taxonomy and phytogeography of bracken – a review. *Bot J Linn Soc* **73**: 1–34

Pamakcu AM and Price JM (1969) Introduction of urinary bladder cancer in rats by feeding bracken fern (*Pteridium aquilinum*). *J Natl Cancer Inst* **43**: 275–281

Pang Y-P and Kozikowski AP (1994) Prediction of the binding site of 1-benzyl-4-((5,6-dimethoxy-1-indanon-2-yl)methyl)piperidine in acetylcholinesterase by docking studies with the SYSDOC program. *J Comput-aided Mol Des* **8**(6): 683–693

Potter DM and Pitman RM (1994) The extraction and characterisation of carcinogens from bracken and the effect of composting. International Bracken Group special publication No. 2, *Bracken: an environmental issue*, Bracken 94, University of Wales, Aberystwyth. **23**: 110–115

Povey AC, Potter D and O'Connor PJ (1996) $^{32}$P-postlabelling analysis of DNA adducts formed in the upper gastrointestinal tissue of mice fed bracken extract or bracken spores. *Br J Cancer* **74**: 1342

Prakash AS, Pereira TN, Smith BL, Shaw G and Seawright AA (1996) Mechanism of bracken fern carcinogenesis: evidence for H-*ras* activation via initial adenine alkylation by ptaquiloside. *Natural Toxins* **4**: 221–227

Rosenberger G (1960) Adlerfarn (*Pteris aquilina*)- die Ursache des sog. Stallrotes der Rinder (Haematuria vesicalis bovis chronica. *Deutsche Tierarztliche Wochenschrift* **8**: 201–208

Saito K, Nagao T, Matoba M, Koyama K, Natori S, Murakami T and Saiki Y (1989) Chemical assay of ptaquiloside, the carcinogen of *Pteridium aquilinum*, and the distribution of related compounds in the pteridaceae. *Phytochemistry* **28**: 1605–1611

© 2000 Cancer Research Campaign 

Saito K, Nagao T, Takatsuki S, Koyama K and Natori S (1990) The sesquiterpenoid carcinogen of bracken fern and some analogues, from the pteridaceae. *Phytochemistry* **29**: 1475–1479

Smith BL (1990) in Bracken Biology and Management, eds. Taylor JA, Smith RT. *AIAS Occasional Publication* **40**: 227–232

Smith BL (1997) The toxicity of bracken fern (genus Pteridium) to animals and its relevance to man. In *Handbook of Plant and Fungal Toxins* D'Mello JPF (ed), pp 63–76. CRC Press: New York

Smith BL, Lauren D, Embling RPP and Agnew MP (1990) Ptaquiloside in Australian and New Zealand ferns as a cause of Oneoplasia. In Bracken Biology and Management. JA Thompson and RT Smith (eds). *Occasional Publication* **40**: 241–246

Smith BL, Embling PP, Lauren DR and Agnew MP (1992) In *Poisonous Plants*, Proceedings of the 3rd International Symposium, Iowa State University 448–452

Smith BL, Seawright AA, Ng J, Hertle AT, Thomas JA and Bostock PD (1994) *Natural Toxins* **2**: 347–353

Swenson DH, Krueger WC, Liu AH, Schpok SL and Li LH (1981) CC-1065: a Novel antitumour agent that interacts strongly with double-stranded DNA. *Proc Am Ass Cancer Res* **22**: 857

Taylor JA (1990) The bracken problem: a global perspective. In Bracken Biology and Management. Taylor JA, Smith RT (eds) pp 3–19. *AIAS Occasional Publication* **40**

Van der Hoeven JCM, Lagerweij WJ, Posthumus MA, Van Veldhuizen A and Holterman HAJ (1983) Aquilide A, a new mutagenic compound isolated from bracken fern (*Pteridium aquilinum*). *Carcinogenesis* **4**: 1587–1590

Villalobos-Salazar J (1985) Carcinogenicidad del *Pteridium Aquilinum* y alta incidencia del cancer gastrico en Costa Rica. *Rev Cost Cienc Med* **6**: 131–139

Villalobos-Salazar J, Menenses A and Sales J (1990) Carcinogenic effects in mice of milk from cows fed on bracken fern, In Bracken Biology and Management, Thomson LA and Smith RT (eds) *AIAS Occasional Publication* **40**: 247–251

Villalobos-Salazar J, Mora J, Menenses A and Pashov B (1995) The carcinogenic effect of bracken spores, presented at *Bracken, an Environmental Issue*, University of Wales, Aberystwyth: 102–103

Wahl AF, Donaldson KL, Fairchild C, Lee FYF, Foster SA, Derners GW and Galloway DA (1996) Loss of normal p53 function confers sensitization to Taxol by increasing G2/M arrest and apoptosis. *Nat Med* **2**: 72–79

Warpehoski MA, Harper DE, Mitchell MA and Monroe TJ (1992) Reversibility of the covalent reaction of CC-1065 and analogues with DNA. *Biochemistry* **31**: 2502–2508

Wells AJ and McNally R (1989), Appraisal of the spatial association of bracken and cancer in England and Wales, presented at *Bracken: An Environmental Issue*, University of Wales, Aberystwyth

Yoshihira K, Fukuoka M, Kuroyanagi M, Natori S, Umeda M, Morohoshi T, Enomoto, M and Saito M (1978) Chemical and toxicological studies on bracken fern *Pteridium aquilinum* var. *latiusculum*. *Chem Pharm Bull* **26**: 2346–2364

 *© 2000 Cancer Research Campaign*
